# Transcriptomic Analyses Reveal the Role of Cytokinin and the Nodal Stem in Microtuber Sprouting in Potato (*Solanum tuberosum* L.)

**DOI:** 10.3390/ijms242417534

**Published:** 2023-12-15

**Authors:** Xia Zhang, Kaien Fujino, Hanako Shimura

**Affiliations:** Graduate School of Agriculture, Hokkaido University, Kita-9 Nishi-9, Kita-ku, Sapporo 060-8589, Japan; zhangxiaycc@gmail.com (X.Z.);

**Keywords:** RNA-seq analysis, potato, sprouting, nodal stem, gibberellin, cytokinin

## Abstract

In potatoes, tuber secondary growth, especially sprouting, deforms the tubers and severely lowers their commercial value. Tuber sprouting is induced by signal substances, such as gibberellin (GA), which are transported to the tuber from the plant body. The molecular mechanism underlying GA-induced sprouting remains ambiguous. Here, we tried to recreate tuber secondary growth using in vitro stemmed microtubers (MTs) (with the nodal stem attached) and MT halves (with the nodal stem entirely removed). Our experiments showed that GA alone could initiate the sprouting of stemmed microtubers; however, GA failed to initiate MT halves unless 6-benzyladenine, a synthetic cytokinin CK, was co-applied. Here, we analyzed the transcriptional profiles of sprouting buds using these in vitro MTs. RNA-seq analysis revealed a downregulation of cytokinin-activated signaling but an upregulation of the “Zeatin biosynthesis” pathway, as shown by increased expression of *CYP735A*, *CISZOG*, and *UGT85A1* in sprouting buds; additionally, the upregulation of genes, such as *IAA15*, *IAA22*, and *SAUR50*, associated with auxin-activated signaling and one abscisic acid (ABA) negative regulator, *PLY4*, plays a vital role during sprouting growth. Our findings indicate that the role of the nodal stem is synonymous with CK in sprouting growth, suggesting that CK signaling and homeostasis are critical to supporting GA-induced sprouting. To effectively control tuber sprouting, more effort is required to be devoted to these critical genes.

## 1. Introduction

Secondary growth is a common issue in crops of potatoes, including sprouting, tuber chaining, and tuber malformations which reduce the tuber starch content, yield, and seed quality, ultimately diminishing commercial value [1]. Potato tubers undergo endogenous dormancy immediately after harvest, which, in fact, parallels tuber enlargement during formation. The apical and subapical meristems (also known as eyes) of tubers become endo-dormant and cannot sprout due to internal physiological factors. Tubers are physiologically competent to sprout after a certain period, usually for one month or more, and finish endogenous dormancy [2,3]. In this article, the term dormancy refers to the endogenous dormant state unless specified otherwise. But before harvest, potato tubers attached to the above-ground part may undergo sprouting called secondary growth, depending on the growing environment. This is thought to be due to the fact that not only the tuber but also the plant body is influenced by the environment. Transporting signal substances to the tubers, such as gibberellin (GA) which is known as a dormancy-breaking hormone, can terminate dormancy and trigger tuber sprouting [4]. Our work reproduced the secondary growth of potatoes using tissue culture and comprehensively analyzed the expression of genes involved in this process.

The dormancy breakage in potato tubers is accompanied by many physiological changes, such as a balance of catabolism, an increase in net DNA and RNA synthesis, and changes in the ratios of abscisic acid (ABA)/GA and ABA/cytokinin (CK) [5,6]. GA has been known to induce growth, and the endogenous GA level increases rapidly toward the end of tuber spontaneous dormancy and the onset of shoot growth [7] by shortening the G1 and S phases of the cells [8] and activating DNA and RNA synthesis [9]. ABA and ethylene suppress tuber sprouting [10]. Ethylene (ET) inhibits tuber sprouting by influencing carbohydrate metabolism, including increased respiration, glucose, total sugar, and reduced fructose and sucrose [11]. ABA inhibits DNA and RNA synthesis to maintain tuber dormancy until the GA/ABA ratio reaches a point favoring GA, promoting cell division and sprouting [12]. *StTCP15* was reported to promote potato tuber sprouting by regulating the dynamic balance of ABA and GA [13], and a high ABA/GA ratio is correlated with dormancy maintenance. In contrast, a low ABA/GA ratio is consistent with dormancy breakage. A low level (<1 mg/L) of auxin application has a stimulating effect on tuber sprouting, but a high dose suppresses tuber sprouting [14,15]. Auxin distribution in tuber buds could affect the order of sprout appearance [16]. CK functions in promoting bud outgrowth emerged decades ago [17,18] and are necessary for bud breakage and initiating tuber sprouting [19] by inducing *CycD3* genes to function during the cell cycle’s G1-S transition (reviewed by Francis, D. et al.) [20]. An abrupt increase in the concentration of endogenous zeatin (naturally occurring CK) and a reduction in ABA at the onset of dormancy breakage have been observed [21]. Both natural (such as zeatin) and synthetic CK (such as kinetin and 6-benzyladenine) applications can rapidly trigger tuber sprouting [22,23]. GA and CK were reported to act synergistically to accelerate sprouting (reviewed by Kolachevskaya et al.) [24]. Remarkably, Hartmann et al. showed that tubers with a decline in CK levels did not respond to GA application by using transgenic potato plants expressing the catabolic cytokinin oxidase/dehydrogenase 1 (*CKX*1) in vitro assay; in contrast, tubers with an increased CK content promoted GA-induced sprouting by harboring the isopentenyltransferase (*IPT*) gene, suggesting that CK could stimulate the dormancy breakage of the excised tuber buds and GA-induced sprouting [25].

Tuberization is a complicated physiological process governed by a few factors, such as the environment, growth regulators, genotypes, photoperiods, temperature, potato cultivars, and sucrose concentration [26]. Microtuber (MT) formation of potatoes is one of the more efficient methods to increase tubers in vitro [27]. MTs could be generated in the axillary bud in a node segment cut from the shoot in short-day or dark conditions, with a high concentration of sucrose and low temperature; treatments with plant growth regulators, like GA inhibitors or ABA [28,29], are also utilized to suppress the activity of GA so that the development can be focused on the swelling of axillary buds (i.e., microtuber formation) rather than shoot growth of axillary buds [30,31]. There is barely any difference between MTs in vitro and average tubers in physiological properties, except for their size and development location, given that tubers form at the tips of the stolons in the field, while MTs are induced in vitro on the medium and form at the axillary buds of explants. Since MTs can sprout in the dark, they serve as an ideal model for studying tuber sprouting under conditions where phytohormones are fed exogenously without photosynthetic involvement. We have studied the effect of some phytohormones co-regulating with GA on the tuber sprouting involved in secondary growth using microtuber halves (hMTs, with the nodal stem entirely removed), whereby we used exogenous auxin, jasmonic acid, ET, and CK to incubate hMTs on the GA-containing medium. Interestingly, GA alone failed to induce the sprouting, and only the CK co-application supported GA in inducing hMTs sprouting.

RNA-seq analysis has been hugely helpful in determining the difference in gene expression, allowing the simultaneous comparison of transcript levels for thousands of genes [32]. Intact sequencing of the potato genome presents an excellent opportunity to identify the genes, enormously improving our understanding of genetic pathways that regulate potato tuber sprouting [33,34]. In the present study, we initiated a comprehensive transcriptome analysis based on the Illumina sequencing platform using potato MTs treated with GA. Exogenous GA-only treatment accelerated tuber sprouting but did not induce dormancy breakage of axillary buds [35], and exogenous CK treatment broke tuber dormancy rapidly and made buds sprout after 2–3 days [22]. However, GA induced stemmed microtubers (stMTs) but not hMTs sprouting, while CK failed in our previous studies. Here, we used GA, CK, both GA and CK (GACK), or no treatment (control) to induce MT sprouting to understand the role of CK and the nodal stem in GA-induced sprouting at the molecular level by screening out genes and profiles with differential expression during tuber eye meristem activation processes (from the breakage of dormancy to the sprouting outgrowth, related to Section 4 Materials and Methods). This study presents the first investigation on the nodal stem (attached with MTs) supporting GA-induced sprouting at the transcriptome sequencing level. Moreover, the results of this study will provide insight into the regulatory mechanisms underlying sprouting in potatoes, and several genes identified will promote further study on controlling tuber sprouting.

## 2. Results

### 2.1. Microtuber Sprouting in Response to Phytohormone Treatment

**Figure 1:**
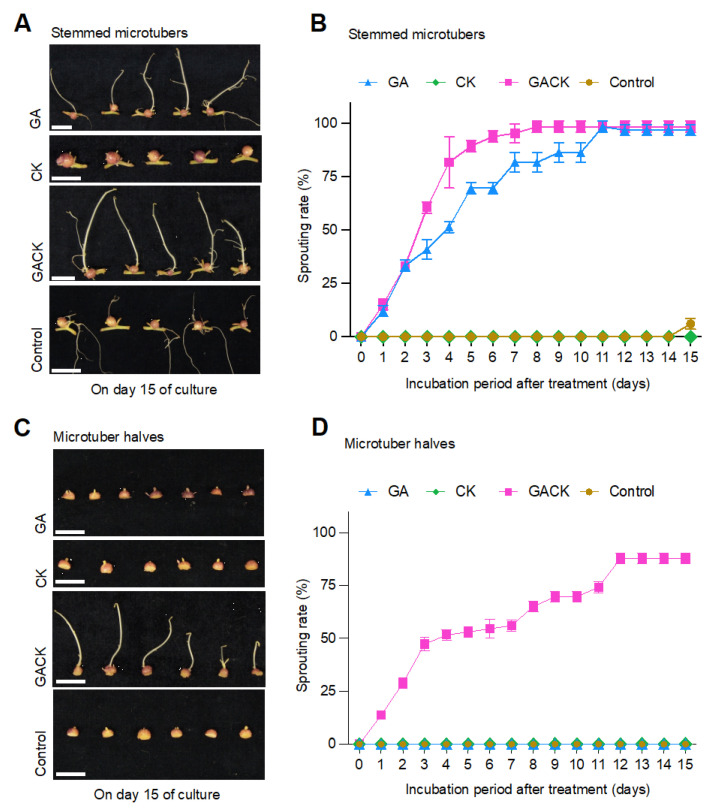
In vitro microtuber (MT) sprouting experiment using 2-week-old potato MTs after incubation with different phytohormone treatments or untreated over the 15-day period. Representative images (**A**) of the visual appearance of sprouting on day 15 of culture and the percentage (**B**) of sprouted MTs attached to the nodal stem (stemmed microtubers, stMTs); representative images (**C**) of the visual appearance of sprouting on day 15 of culture and the percentage (**D**) of sprouted MTs cut in half along the equatorial plane and with the nodal stem entirely removed (microtuber halves, hMTs). MTs were cultured with 10 μM GA_3_ (GA), 10 μM 6-benzyladenine (CK), both GA and CK (GACK), or untreated (control), respectively. Each value represents the mean ± standard deviation of three replicates (n = 30). Bar = 10 mm.

**Figure A1:**
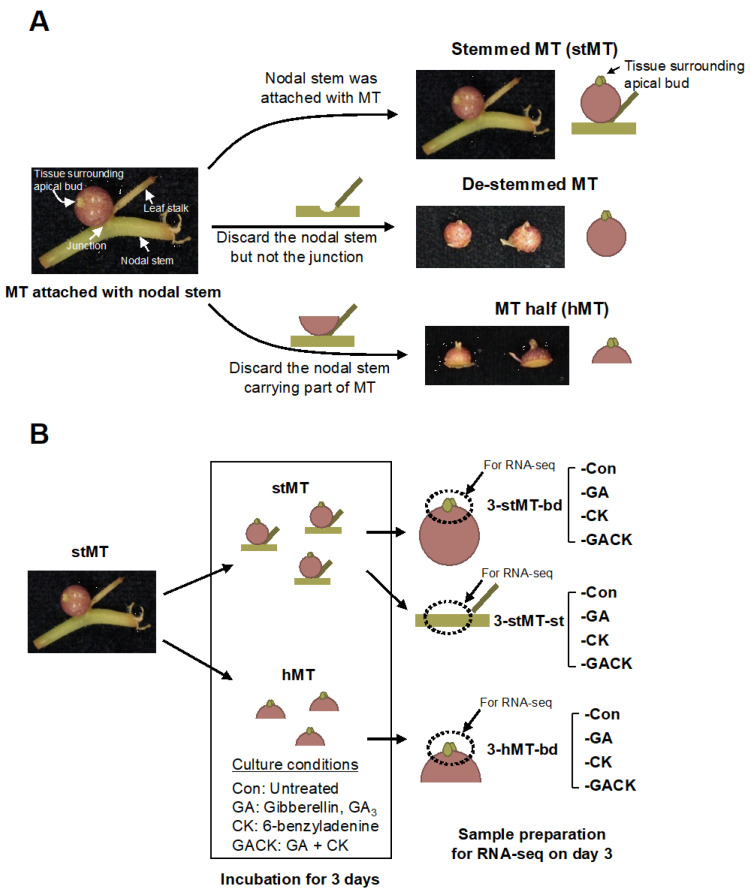
Schematic representation of microtuber (MT) treatment in this study. (**A**) Three preparations were used to monitor sprouting: (1) the nodal stem was attached to the MT (stemmed MT, stMT), (2) the stem piece was removed, but the junction remained in the MT (destemmed MT), and (3) the nodal stem was completely removed, including the junction in the microtuber (microtuber half, hMT). (**B**) Sample preparation for RNA-seq: the black dotted ellipses represent the sampled part for RNA extraction. For 3-stMT-bd-GA, -CK, -GACK, or -Con, the apical bud from stMT on day 3 of culture by GA, CK, GACK, or untreated Con; for 3-stMT-st-GA,-CK, -GACK, or -Con, the nodal stem with junction part from stMT on day 3 of culture by GA, CK, GACK, or culture by Con; for 3-hM-bd-GA, -CK, -GACK, or -Con, the apical bud from the MT half (hMT) on day 3 of culture by GA, CK, GACK, or untreated Con.

To understand the effect of the nodal stem on sprouting, we investigated microtuber (MT) sprouting using three kinds of MTs: stMTs, destemmed microtubers (deMTs), and hMTs, as described in Section 4 Materials and Methods. The detailed preparation for in vitro MTs is depicted in the schematic drawing of Appendix A, Figure A1A. We did not observe any sign of sprouting among the three kinds of MTs on the control medium until day 15 of culture when about 6% of stMTs sprouted, so we considered 15 days as the rest period for MTs in the dark at 20 °C in our study and stopped observing the sprouting behavior after day 15 (Figure 1). The sprouting rate is the percentage of sprouted microtubers. The apical bud of the dormant microtuber is invisible, surrounded by yellow-green tissues. Once the dormancy is broken, the apical bud (i.e., sprout) emerges from the center of these yellow-green tissues. In our observation, the microtuber is considered as sprouting when the emerged sprout has 2 mm of length at the morphological level, given that apical buds may grow temporarily but never elongate more than 2 mm. The sprouting process of MTs for 15 days is shown in Figure 1 and Supplementary Figure S1.

For the stMTs, we observed that the sprouting behavior occurred after GA treatment alone and after both GA and CK treatment (GACK) (Figure 1A). There was barely a difference for shoots on day 15 of culture between GA and GACK treatments at the morphological level. We did not observe any sign of sprouting behavior with CK treatment alone after 15 days. GA and GACK treatment could induce sprouting on day 1 (Figure 1B), reaching about a 55% and 86% sprouting rate on day 4 of culture, respectively, indicating that exogenous GA has a significant effect on promoting stMTs sprouting growth and that exogenous CK co-application enhances the sprouting behavior. There was no significant difference in sprouting rate between the GA and GACK treatments, as shown by the adjusted *p* value > 0.05 of Tukey’s multiple comparisons test result (Supplementary Table S1). During observation, deMTs sprouted similarly to stMTs until day 4 of culture, when they stopped sprouting after reaching 34%, suggesting that the junction part (between the MT and the nodal stem) could support GA-induced sprouting like the nodal stem, but it was inadequate for subsequent sprouting. However, the CK co-application could make up for the insufficiency; about 71% of MTs sprouted on day 15 of culture with GACK treatment. There were significant differences in sprouting rate between the GA and GACK treatments with the adjusted *p* value (0.003) < 0.05 of Tukey’s multiple comparisons test result (Supplementary Figure S1A,B, Table S1) due to the CK co-application.

We noticed that the sprouting behavior in hMTs only occurred after GACK treatment (Figure 1C,D) when the nodal stem was excised, suggesting that both GA and CK are essential for MT sprouting, and that GA alone or CK alone is insufficient to induce the sprouting behavior. When treated with GACK, there were noticeable differences in shoot morphology between stMTs and hMTs on day 10 of the culture (Supplementary Figure S1A). However, this difference disappeared on day 15 of the culture. In addition, we conducted a Tukey’s multiple comparisons test in sprouting rate among three sprouting-induced conditions using one-way ANOVA with the adjusted *p* value > 0.05; there were no significant differences in the sprouting behavior among GA-treated and GACK-treated stMTs sprouting, and GACK-treated hMTs (Supplementary Table S1). These results indicate that the nodal stem or exogenous CK co-application is essential for supporting GA to induce MT sprouting, and there was no significant difference in sprouting-induced conditions between GA combined with the nodal stem and GA combined with CK co-application.

### 2.2. Transcriptome Sequencing and De Novo Assembly

In this study, we used *Solanum tuberosum* L. subsp. *andigena* (2n = 4x = 48) for the research. For the transcriptome analysis, 36 samples generated 103.61Gb of clean data through RNA sequencing. About 92.09–93.58% of clean data achieved a quality score of Q30 (sequencing error rate < 0.1%), and 84.96–89.14% of the clean reads were successfully mapped to the potato reference genome (S. tuberosum PGSC_DM_v4.03) [30] (Table 1), indicating that all libraries were of high quality. Based on gene expression, principal component analysis (PCA) showed that the 36 samples were assigned to three groups, each associated with one tissue type from two kinds of MTs. As expected, no spurious sample clusters were identified. The first (PC1) and second (PC2) principal components accounted for 34.06% and 10.15% of the total variance, respectively. PC1 separated samples into two big groups corresponding to tissue differences between tissue types: apical bud tissues of stMTs and hMTs, and nodal stem tissues of stMTs. In comparison, PC2 separated samples into two big groups corresponding to the difference between MT types: stMTs including apical bud tissues and nodal stem tissues, and hMTs including apical bud tissues. The PC1 value was greater than PC2 value, demonstrating that gene expression patterns were more affected by different tissue types than MT types. Moreover, gene expression data from the same type of tissue clustered together but differed among the treatments with different phytohormones (Supplementary Figure S2). The first and second principal components accounted for 34.06% and 10.15% of the total variance, respectively, showing a good correlation among the three biological replicates for the 36 samples of the RNA-seq results.

### 2.3. Differential Transcriptome Response to Exogenous Hormone Treatments

We first calculated the differentially expressed genes (DEGs) using DESeq2 for nine pairwise comparisons between the phytohormone treatment (GA, CK, or GACK) and the untreated control (Con) in the apical buds of stMTs or hMTs or in the nodal stems of stMTs cultured on day 3 (3-), respectively (Supplementary Figure S3). For the apical buds of stMTs (3-stMT-bd), 2789 DEGs were detected after treatment with GA when compared to Con (3-stMT-bd-GA/Con), 698 DEGs were detected after treatment with CK when compared to Con (3-stMT-bd-CK/Con), and 1811 DEGs were detected after treatment with GACK when compared to Con (3-stMT-bd-GACK/Con). For the apical buds of hMTs (3-hMT-bd), 2095, 1667, and 4058 DEGs were detected in the comparisons of 3-hMT-bd-GA/Con, 3-hMT-bd-CK/Con, and 3-hMT-bd-GACK/Con, respectively. For the nodal stems of stMT (3-stMT-st), 239, 516, and 850 DEGs were detected in the comparison of 3-stMT-st-GA/Con, 3-stMT-st-CK/Con, and 3-stMT-st-GACK/Con, respectively. Excluding duplicates, a total of 6515 DEGs were detected. The amount of DEGs were much larger in both 3-stMT-bd-GA/Con and 3-hMT-bd-GACK/Con, indicating that the combination of GA and the nodal stem, and the combination of GA and CK treatments play a vital role in this sprouting behavior.

To better understand the difference in the transcriptional response of sprouting to exogenous hormone treatment, we performed DEG annotation and functional categorization using Gene Ontology (GO) and KEGG pathway enrichment analysis for 6515 DEGs (Supplementary Table S2). GO enrichment analysis has three major categories: biological processes, cellular components, and molecular functions. This study mainly focused on biological processes to identify the vital processes in sprouting behavior. Interestingly, we detected that several hormone-related terms associated with auxin, including “response to auxin”, “auxin polar transport”, “auxin efflux”, and “auxin-activated signaling pathway”, were significantly enriched in biological processes. This result indicates that endogenous auxin responded to hormone treatment. Next, KEGG pathway enrichment analysis showed that genes were enriched into sixteen pathway terms (*p* < 0.05). Of the sixteen terms, “Plant hormone signal transduction” (sot04075) was the central pathway with the lowest *p* value. Notably, a CK-related pathway, “Zeatin biosynthesis” (sot00904), was also significantly enriched. These results suggest that terms like “response to auxin”, “Plant hormone signal transduction”, and “Zeatin biosynthesis” should be the main focus of this transcriptome analysis.

### 2.4. Cytokinin- and Auxin-Activated Signaling Are Involved in Microtuber Sprouting

**Figure 2:**
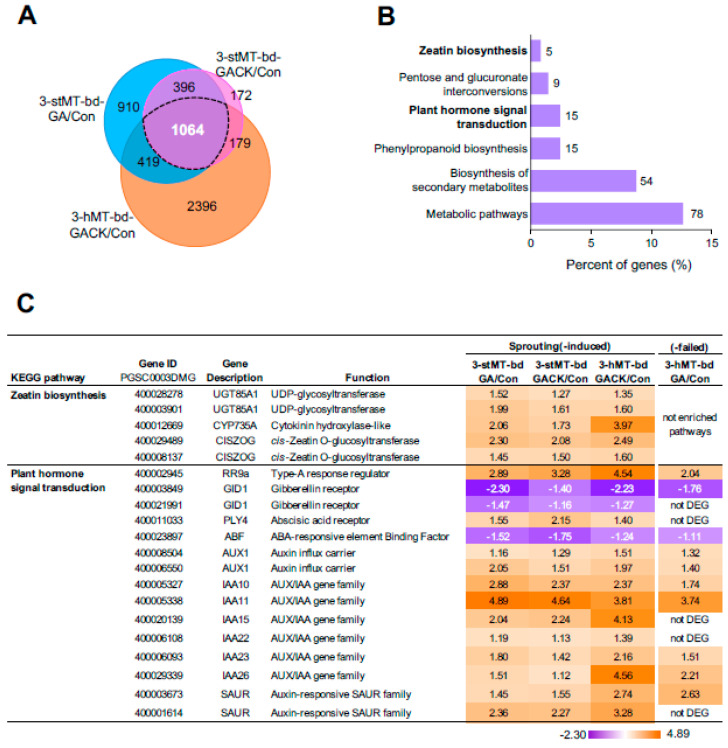
Differentially expressed genes (DEGs) identified in apical bud tissues (bd) of MTs in sprouting-inducible conditions. (**A**) A Venn diagram to visualize the similarities and differences in DEGs among three comparisons: 3-stMT-bd-GA vs. Con (3-stMT-bd-GA/Con), 3-stMT-bd-GACK/Con, and 3-hMT-bd-GACK/Con. The 1064 DEGs of overlap in the diagram represent genes correlated with each respective group of genes. (**B**) Enriched KEGG pathways for the 1064 shared DEGs analyzed by DAVID (*p* < 0.05), visualized using GraphPad PRISM. Counts of DEGs included in each KEGG pathway are shown next to the bar in the graph. (**C**) The table shows genes included in the two pathway terms (“Zeatin biosynthesis” and “Plant hormone signal transduction”) among four comparisons, 3-stMT-bd-GA/Con, 3-stMT-bd-GACK/Con, 3-hMT-bd-GACK/Con, and 3-hMT-bd-GA/Con, with a heatmap showing gene expression levels as log2 Fold Change (FC). Orange represents upregulation, and purple represents downregulation compared to control (untreated).

To understand the detailed gene response to sprouting growth, we compared the differences in DEGs in apical bud tissue among the three sprouting-inducible conditions (stMTs treated with GA or GACK, or hMTs treated with GACK) with the untreated Con: 2789, 1811, and 4058 DEGs in comparison to 3-stMT-bd-GA/Con, 3-stMT-bd-GACK/Con, and 3-hMT-bd-GACK/Con, respectively (Supplementary Figure S3, Figure 2A). They shared 1064 DEGs and harbored 910, 172, and 2396 unique DEGs, respectively. The largest number (4058) of DEGs were detected in the GACK-treated apical bud tissues of hMTs, followed by 2789 DEGs detected in the GA-treated apical bud tissues of stMTs, suggesting that this sprouting behavior was mainly in response to GA combined with CK and GA combined with the nodal stem. To reveal the molecular similarities in this sprouting behavior, we analyzed the 1064 shared DEGs. KEGG pathway enrichment analysis revealed that six pathway terms were significantly enriched (*p* < 0.05) (Figure 2B). In the “Plant hormone signal transduction” pathway (Figure 2C), fifteen genes were grouped: one was type A response regulator *RR9a* involved in the negative regulation of cytokinin signaling; two were ABA-related genes, *PYL4* (ABA core regulator) and *ABF* (annotated as ABA-responsive bZIP transcriptional factor); two were GA receptors, *GID1*; and ten were auxin-related genes, *AUX1* (auxin influx carrier), *Aux/IAA*, and *SAUR* (small auxins upregulated by RNA, auxin early-responsive genes). Out of the fifteen genes, only three were downregulated: *GID1*s and *ABF*. In contrast, genes related to CK, auxin, and *PYL4* were upregulated. These results suggest that genes related to CK and auxin are critical in sprouting growth. Five genes were detected in the “Zeatin biosynthesis” pathway: two were cis-zeatin (cis-CK) O-glucosyltransferase (*CISZOG*) involved in the synthesis of cis-zeatin riboside; two were UDP-glycosyltransferase, *UGT85A1*, associated with the process of CK homeostasis; and one was *CYP735A*, annotated as cytokinin trans-hydroxylase, involved in trans-zeatin synthesis. This suggests that the expressions of genes related to CK homeostasis and signaling are enhanced in sprouting growth.

**Figure 3:**
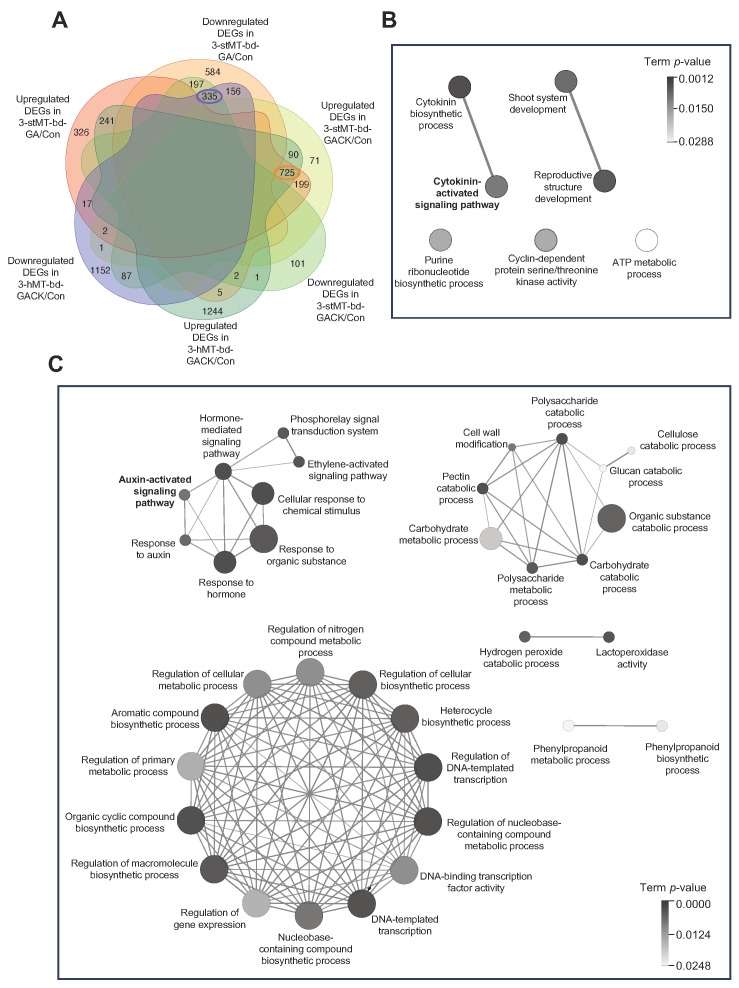
DEGs identified in apical bud tissues (bd) of MTs in sprouting-inducible conditions. (**A**) A detailed Venn diagram to visualize the similarities and differences in DEGs among three sprouting-induced comparisons: 3-stMT-bd-GA vs. Con (3-stMT-bd-GA/Con), 3-stMT-bd-GACK/Con, and 3-hMT-bd-GACK/Con. Here, 335 and 725 DEGs of overlap in the diagram represent co-downregulated and co-upregulated genes, respectively. Network analysis of enriched GO terms for 335 (**B**) and 725 DEGs (**C**), respectively, and significantly enriched GO terms (*p* < 0.05) were used to visualize the network using ClueGO plugin in Cytoscape (v3.10.0). The related terms sharing similar associated genes were fused to reduce redundancy using kappa statistics. The size of each circle represents gene numbers in each process.

Of the 1064 shared DEGs, 335 genes were downregulated and 725 were upregulated (Figure 3A), suggesting that the activation of genes played a more critical role in responding to this sprouting behavior. We next performed GO analysis for both groups independently to reveal the molecular difference between the up- and downregulated common DEGs in sprouting buds (Figure 3B,C). ClueGO network analysis showed that seven significantly enriched GO terms classified into five groups were detected for 335 DEGs using GO term fusion with the criteria of *p* value < 0.05 (related to Section 4 Materials and Methods). The most significantly enriched terms (with the lowest *p* value) were summarized as the cytokinin biosynthesis process, including cytokinin-activated signaling pathway, shoot system development, purine ribonucleotide biosynthesis, cyclin partner regulatory, and chemical reactions and pathways. During cell development’s proliferation and expansion stages, CK is known to promote cell division and increase cell expansion. The downregulation of genes related to cytokinin biosynthesis may result in the cells ceasing to develop and grow. Therefore, the co-application of exogenous CK is necessary to ensure the sprouting of apical buds.

On the other hand, thirty-five significantly enriched GO terms clustered into five groups were detected for 725 DEGs using GO term fusion with the criteria of *p* value < 0.05. The highest overrepresented GO term (with the lowest *p* value) was “response to hormone” including auxin-activated signaling pathway, followed by DNA-binding transcription factor activity, organic substance catabolic process, hydrogen peroxide catabolic process, and phenylpropanoid metabolic process, suggesting that the regulation of transcription activity and chemical reactions resulting in the breakdown of organic substances and hydrogen peroxide are essential for regulating the state or activity of organisms during the development of sprouting, including hormone-induced signaling. These results gave a preliminary recognition of gene expression changes in this sprouting behavior, showing that genes related to shoot formation associated with CK-activated signaling were downregulated, while genes related to regulating the organism’s state associated with auxin-activated signaling were upregulated during sprouting growth. The detailed genes involved in each term are filed in Supplementary Table S3.

### 2.5. The Effect of GA on Microtuber Sprouting in the Absence of the Nodal Stem

Interestingly, when we performed KEGG and GO enrichment analysis for 3-hMT-bd-GA/Con (Supplementary Table S4), we found that the “Zeatin biosynthesis” pathway was not enriched in GA-treated hMT (the right-most column, “Sprouting-failed”, in Figure 2C), but the “Plant hormone signaling transduction” pathway was enriched; in this pathway, twenty-two genes were enriched, and seven of them were auxin-related genes, which also were detected in sprouting-induced conditions. They had the exact expression change (up- or downregulated) as that in sprouting-induced conditions. Only four genes, *PLY4*, *IAA15*, *IAA22*, and *SAUR50*, were detected explicitly in sprouting buds, reflecting the significance of the upregulation of *PLY4*, *IAA15*, *IAA22,* and *SAUR50* in response to sprouting. Notably, GO terms showed that “response to auxin” connected with the “auxin-activated signaling pathway” was also enriched, and genes related to this term were all downregulated (Supplementary Table S4). According to the results, GA can activate auxin signaling even in failed sprouting conditions. However, GA treatment failed to change the expression of genes related to the “Zeatin biosynthesis” pathway, *PLY4* (one ABA core regulator), and some auxin-related genes (such as *IAA15*, *IAA22*, and *SAUR50*) under conditions without the nodal stem. These results suggest that the lack of regulation for genes related to the pathway and *PLY4, IAA15*, *IAA22*, and *SAUR50* could explain why GA fails to induce sprouting without the support of the nodal stem. This indicates that the auxin-related *IAA15*, *IAA22*, and *SAUR50,* the ABA-related *PLY4*, and the “Zeatin biosynthesis” pathway-related *CYP735A*, *CISZOG,* and *UGT85A1* play a vital role during sprouting growth.

### 2.6. The Effect of the Nodal Stem on GA-Induced Microtuber Sprouting

**Figure 4:**
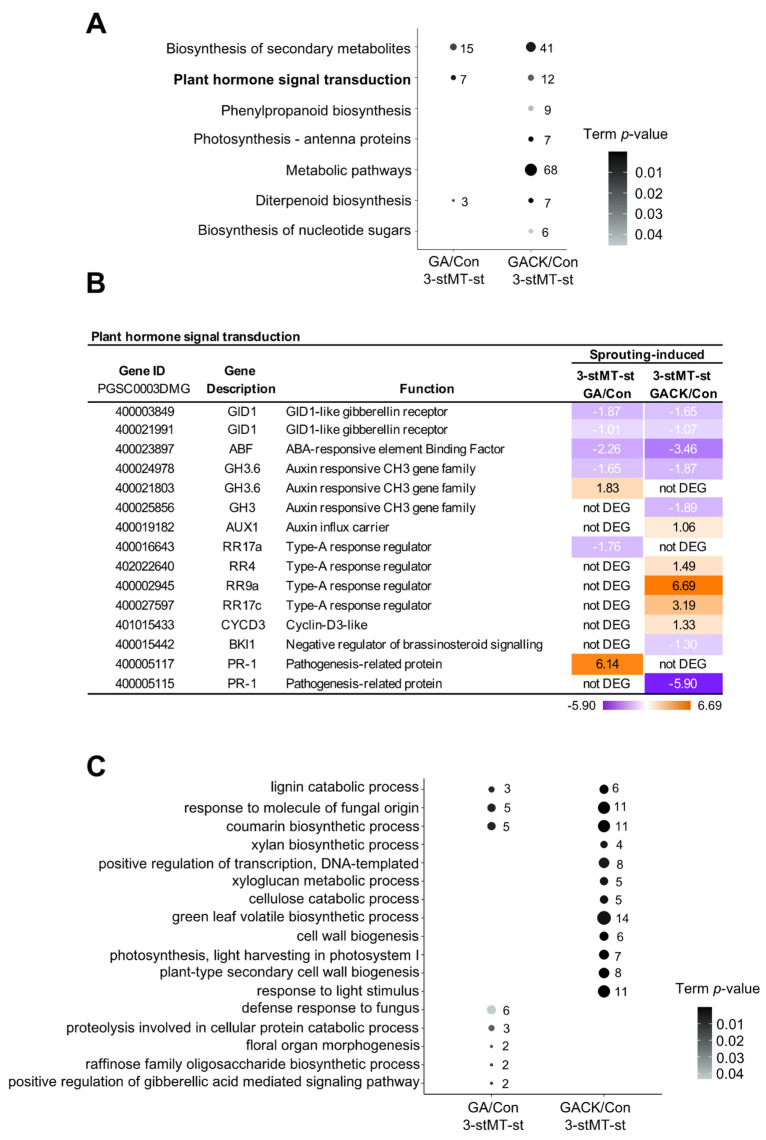
Functional annotations of DEGs in the nodal stem on day 3 of culture with exogenous GA and GACK, respectively. A comparison of enriched KEGG pathways (**A**) and GO terms in biological processes (**C**) between two comparisons of phytohormone treatment (GA or GACK) with control (Con, untreated) in the nodal stem of stMT using DAVID (*p* < 0.05), visualized using the ggplot2 package in R. Counts of DEGs included in each KEGG pathway are shown next to the plot. (**B**). The table shows genes included in the pathway “Plant hormone signal transduction” for each comparison, with a heatmap showing gene expression levels as log2 FC. Orange represents upregulation, and purple represents downregulation compared to control (untreated).

Given that the nodal stem (-st) attached to MTs might play a vital role in this sprouting behavior, we first compared gene expression changes in the nodal stem involved in sprouting on day 3 of culture between GA and GACK treatment. KEGG enrichment analysis (Figure 4A) showed that they shared three pathways. Genes related to the GA receptor (*GID1*) and ABA-responsive bZIP transcriptional factor (*ABF*) were downregulated in both comparisons. Three genes involved in CK negative response regulators (*RR4*, *RR9a*, and *RR17a*) were upregulated in 3-stMT-st-GACK/Con. However, *RR17c* was downregulated in 3-stMT-st-GA/Con, indicating that the activation of CK is involved in the nodal stem due to the negative regulation of *RR17c*; one *PR-1* (400005117) and *GH3.6* (400021803) (an auxin inactivation gene) were uniquely upregulated (Figure 4B) in 3-stMT-st-GA/Con, reflecting that the reduction in auxin biosynthesis signaling may be involved in GA-treated nodal stems, given that the enhanced expression of *StPR1* could downregulate the expression of *StYUC1* (an auxin synthesis gene) and upregulate *StGH3.6* in potatoes [36]. In contrast, another *PR-1* (400005115) and *GH3.6* (400024978) were downregulated in 3-stMT-st-GACK/Con, probably because the exogenous CK co-application led to a negative feedback regulatory loop via endogenous CK biosynthesis, which finally influenced the expression of genes related to auxin and CK. GO enrichment analysis showed that they shared three GO terms in the biological process (Figure 4C), and the “positive regulation of gibberellic acid induced signaling pathway” term was only enriched in 3-stMT-st-GA/Con. These results indicate that treatment with GA alone induced the changes in CK-related gene expression, and CK signaling was inhibited when CK was co-applied in the presence of the nodal stem.

To examine the effect of stem excision on the expression of genes, we next focused on the differences in DEGs detected in 1512 and 818 unique DEGs harbored by 3-stMT-bd-GA/Con and 3-hMT-bd-GA/Con, respectively (Supplementary Figure S4A). They shared three KEGG pathway terms (Supplementary Figure S4B), including the “Plant hormone signal transduction” pathway. Eighteen genes were grouped together for the 1512 unique DEGs. Nine genes were related to auxin, four genes were associated with ABA, three were *PLY4*, and one was *PP2*C (the regulator for ABA signaling) (Supplementary Figure S4C). On the other hand, nine genes were detected for the 818 unique DEGs. Four genes were related to auxin, such as *ARF5* and *ARF9*, no *Aux/IAA* genes were detected, and two genes were related to CK, *RR9* and *PHP4b* (known to suppress CK signaling) (Supplementary Figure S4C). This comparison suggests that the expression level of CK-, auxin-, and ABA-related genes changes in response to GA-involved MT sprouting in the presence of the nodal stem.

### 2.7. The Effect of CK on Microtuber Sprouting

Notably, GA can induce hMTs sprouting when exogenous CK is co-applied (Figure 1C,D). To examine the effect of the existence of CK on the expression of plant hormone-related genes, we next compared DEGs between 2428 and 465 unique DEGs harbored by 3-hMT-bd-GACK/Con and 3-hMT-bd-GA/Con, respectively (Supplementary Figure S5A). No KEGG pathways were significantly enriched for the 465 unique DEGs, and nine KEGG pathways were enriched for the 2428 unique DEGs (Supplementary Figure S5B). Twenty-four genes were included in the “Plant hormone signal transduction” pathway; fifteen were related to auxin, five genes were associated with CK, and there was one ABA core regulator *PLY4*. This comparison shows that the expression level changes in CK- and auxin-related genes are vital in sprouting.

KEGG enrichment analysis was used to determine the detailed genes associated with CK treatment, which failed to induce MT spouting. The result showed that the “Plant hormone signal transduction” pathway was shared by 3-stMT-st-CK/Con and 3-hMT-bd-CK/Con (Supplementary Figure S6A). The genes related to the GA receptor, such as *GID1*, were not enriched, suggesting that CK treatment could not change the expression of genes related to GA. This may explain why CK failed to cause signs of sprouting, given that GA is a negative regulator of potato tuberization and that bioactive GA levels are rapidly reduced in stolons with the onset of tuberization. Genes related to the CK negative receptor were upregulated. Four auxin-related genes (*AUX1* and *IAA26*) were upregulated (Supplementary Figure S6B) which were also detected in sprouting-induced conditions (Figure 2C). Strikingly, the regulation of *IAA23* was in reverse to that detected in sprouting-induced conditions. GO enrichment analysis showed that three comparisons shared no terms in the biological process (Supplementary Figure S7, Table S5), and the term “response to auxin” was significantly enriched in 3-stMT-st-CK/Con and 3-hMT-bd-CK/Con. Genes involved in this GO term are mostly downregulated *SAURs*, compared with the genes detected in response to sprouting. However, *SAUR50* was not detected as DEGs. Moreover, no DEGs related to GA were detected in CK-treated conditions, indicating that although CK treatment may be involved in inhibiting genes related to auxin, the lack of regulating *SAUR50* and genes related to GA made CK fail to induce sprouting.

### 2.8. Weighted Gene Co-Expressed Gene Network Analysis (WGCNA)

**Figure 5:**
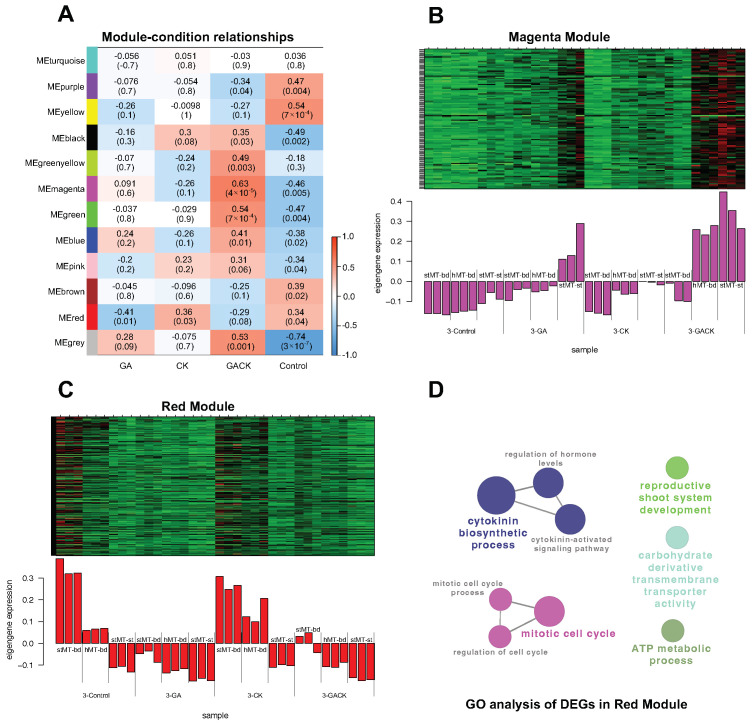
A weighted gene co-expression network was established for a total of 6515 DEGs detected in this work. (**A**) Module–trait associations based on Pearson correlations. Each row represents one module, and the gene numbers in each module are shown in Supplementary Table S6. Expression patterns of genes are in magenta (**B**) and red modules (**C**). (**D**) Network analysis of enriched GO terms (*p* < 0.05) for 275 DEGs in the red module visualized with the ClueGO plugin in Cytoscape (v 3.10.0). The color and size of each circle represent the group and significance (*p* value) of each term, respectively.

To further investigate the gene regulatory network of phytohormone signaling involved in this sprouting behavior, we employed a weighted gene co-expression network analysis (WGCNA) using a total of 6515 DEGs. The analysis resulted in 12 co-expression modules labeled and highlighted using different colors (Figure 5A, Supplementary Table S6). The results revealed that the magenta module (r = 0.63, *p* value = 4 × 10^−5^) was highly and positively correlated with GACK-triggered gene expression. In this gene set (117 genes), genes involved in the nodal stem of stMTs treated with GA and GACK (Figure 5B) were highly upregulated in this module, according to the kME value (eigengene-based connectivity) value. KEGG enrichment analysis showed that genes related to only CK and auxin were enriched in the “Plant hormone signal transduction” pathway (Supplementary Table S7), including *RR4* and *SAUR 71*. These results suggest that endogenous CK was involved in the nodal stem, given that the upregulation of DEGs relevant to sprouting were detected in the nodal stem with GA-only treatment. In contrast, the red module (r = −0.41, *p* value = 0.01) had the highest negative correlation with GA-triggered gene expression. In this gene set (275 genes), genes involved in the nodal stem and apical buds of stMTs and in the apical buds of hMTs treated with GA and GACK (Figure 5C) were highly downregulated in this module according to the kME value (Supplementary Table S7). GO enrichment analysis revealed that genes involved in the “cytokinin biosynthetic process” connecting with the “cytokinin-activated signaling pathway” were downregulated (Supplementary Table S8), reflecting that GA could result in the inactivation of genes related to CK biosynthesis because DEGs in this term were downregulated even in sprouting-failed buds of hMTs with GA-treatment. These results further suggest that treatment with GA alone could cause a reduction in gene expression relevant to CK in apical buds, and the nodal stem might be a source of endogenous CK for GA-induced sprouting.

### 2.9. Correlations of RT-qPCR and RNA-Seq Datasets

**Figure 6:**
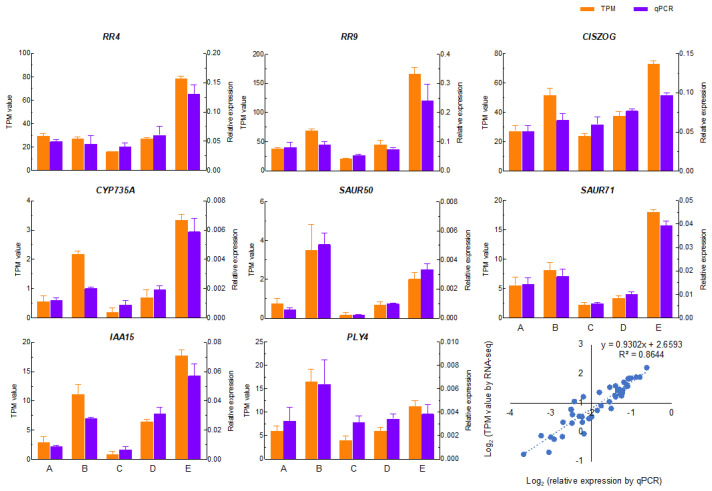
Correlation of expression patterns of selected genes from RNA-Seq and qRT-PCR. Eight genes were selected for transcriptomic and qPCR analysis using apical bud tissues (bd) of the stemmed microtubers (stMTs) and microtuber halves (hMTs) on day 3 of culture treated with GA, GACK, and control (untreated) (related to Section 4 Materials and Methods and Section 2 Results sections). TPM values were obtained after mapping RNA-seq reads to reference genes (Gene: PGSC0003DMG_). qPCR (real-time PCR) data of three replicates are shown as mean (±SE). *RR4*: 402022640; *RR9*: 400003084; *CISZOG*: 400028331; *CYP735A*: 400012669; *SAUR71*: 400001655; *SAUR50*: 400001614; *IAA15*: 400020139; *PYL4*: 400011033. A, B, C, D, and E represent 3-stMT-bd-Con, 3-stMT-bd-GA, 3-hMT-bd-Con, 3-hMT-bd-GA, and 3-hMT-bd-GACK.

RNA-seq analyses showed that the lack of regulation for auxin-related *IAA15*, *IAA22*, and *SAUR50*; CK-related *CYP735A*, *UGT85A1*, and *CISZOG*; and ABA-related *PLY4* may be responsible for GA failure to induce sprouting in the absence of the nodal stem. We next selected eight relevant genes to verify the RNA-seq expression profile associated with GA treatment (Figure 6), including *IAA15*, *SAUR50*, *CYP735A*, *CISZOG*, and *PLY4,* with the criterion of the higher TPM value in RNA-seq results. Additionally, we also selected other CK-related genes, *RR4* and *RR9*, and auxin-related *SAUR 71*, given that these three genes were the hub genes enriched in “plant hormone signaling transduction” of 117 genes set in magenta module of WGCNA (Supplementary Table S7). qPCR was tested on the apical bud tissue’s gene expression in GA-treated stMTs and hMTs, and GACK-treated hMTs. The results showed that despite variations between RNA-seq data and qPCR results regarding the relative expression of the selected genes, the linear regression analysis indicated a high correlation (R^2^ = 0.8644), demonstrating that the expression profiles of the selected genes were broadly consistent with data derived from the RNA-Seq analysis. This suggests that the transcriptome results were highly reliable and repeatable.

## 3. Discussion

### 3.1. Microtuber Sprouting in Response to Phytohormone Treatment

It has been known for many years that the exogenous GA promotes tuber sprout growth rather than dormancy release [7]. However, the ability of GA to induce sprouting remains controversial. In this study, we used 10 μM GA_3_ (GA) to induce MT sprouting. The GA-only application induced the sprouting of both stMTs and deMTs (Figure 1A,B, Supplementary Figure S2), but it failed to terminate the dormancy of hMTs (Figure 1C,D). Interestingly, the hMTs recovered and sprouted after the exogenous CK was co-applied. Although the shoot growth of hMTs was not as fast as that treated with GA in stMTs at the beginning, their elongation levels matched on day 15 of culture (Figure 1A,C), agreeing with the report that GA has a dormancy-terminating capacity but requires CK to induce sprouting growth in tubers [25]. CK has been known as the only chemical to release axillary buds from dormancy and is locally biosynthesized in the nodal stem and transported into axillary buds to promote bud outgrowth after decapitation [37]. In the magenta module of the WGCNA results, genes related to CK in the “plant hormone signaling pathway” were positively regulated in the nodal stem of stMTs with GA alone treatment (Figure 5B, Supplementary Table S7), suggesting that endogenous CK was involved in the nodal stem (Figure 5B) in response to sprouting due to the fact that the GA treatment resulted in the downregulation of genes involved in the “cytokinin-activated signaling pathway” (Figure 3B and Figure 5C and Supplementary Table S7). This makes the nodal stem necessary to help GA induce MT sprouting. This may be because we cultured MTs on a medium using a high level of sugar in the dark, leading to CK accumulation in the nodal stem. This assumption is supported by studies showing that sugars can promote CK synthesis and accumulation to trigger the induction of bud outgrowth in rose, potato stems, and Arabidopsis shoots [38,39].

Furthermore, previous studies reported that the effect of CK on tuber dormancy may be due to experimental systems, substances, or potato cultivars [40,41]. Our study showed that the CK-only application failed to cause any signs of sprouting for 15 days, except for some tissue surrounding the apical bud, unless exogenous GA was also applied, agreeing with the result of Hartmann et al. [25]. These results further led us to infer that GA alone or CK alone is inadequate to initiate MT sprouting. Both are essential for sprouting.

### 3.2. Regulation of the Phytohormone Signaling Response to Microtuber Sprouting

WGCNA results demonstrated that the GA-only application significantly downregulated genes involved in the “cytokinin-activated signaling pathway” (Figure 5C), including genes in the apical bud of hMTs treated with GA, which failed to induce sprouting. Zhuang et al. reported that the application of GA failed to induce bud outgrowth through an antagonistic interaction with CK because the GA decreased the endogenous CK content [42]. However, our network analysis showed that gene expression in the “cytokinin-activated signaling pathway” and “auxin-activated signaling pathway” was induced and enhanced, respectively, during sprouting growth (Figure 3B,C). These results indicate that the GA-only application can cause a reduction in “cytokinin-activated signaling”, but CK and GA have been known to act synergistically to accelerate potato sprouting [24], which may explain why GA requires CK to initiate sprouting. However, this reduction in CK-activated signaling may be caused by auxin involvement, which was induced by GA application.

The elevated auxin level would reduce the active CK content in potatoes [43]. Our RNA-seq analysis showed that GA alone elevated the auxin level by upregulating the expression of genes involved in the “auxin-activated signaling pathway”, connecting with the “response to auxin” of enriched GO terms for 3-hMT-bd-GA/Con (Supplementary Table S4). CK can biosynthesize de novo locally in the nodal stem to promote axillary bud outgrowth, but auxin suppresses the local biosynthesis of CK [37] by negatively regulating the expression of the adenosine phosphate-isopentenyltransferase (*IPT*) gene, which encodes a key enzyme in CK biosynthesis [44]. Agreeing with that, *StIAA15* and *StIAA22* were reported to be highly expressed in potato tuber sprouts [45], and our results showed that the gene expression of *IAA15* and *IAA22* were only increased in sprouting conditions (Figure 2C, Supplementary Figures S5C and S6B). Buskila et al. found that NAA (a synthetic auxin) treatment suppressed the promotive effects of GA and CK on the bud elongation of tubers [46]. These results indicate that the GA application might activate auxin-activated signaling, resulting in a repression of CK-activated signaling, which makes it even more necessary for CK to help GA induce MT sprouting in this study. These results allowed us to conclude that GA application elevated auxin levels, reducing CK content. The levels of endogenous auxin and CK content during sprouting require further investigation with a time course design to understand the interaction among GA, CK, and auxin in this sprouting behavior.

RNA-seq analysis also revealed that an ABA core regulator, *PYL4*, was upregulated in sprouting conditions but not detected as DEGs in sprouting-failed conditions (Figure 2C). *PYLs* function at the first step of the ABA signal pathway, activating the downstream ABA signaling cascade [47] and playing a primary role in seed dormancy and germination [48]. Notably, the downregulation of *PP2C* was only detected in 1512 unique DEGs in 3-stMT-bd-GA/Con (Supplementary Figure S4). In the absence of ABA, *PYLs* cannot bind to *PP2C,* and therefore *PP2C* activity is high, which prevents the phosphorylation and activation of SnRK2s and downstream substrates, such as ABA-responsive element-binding factors (*ABFs*) [49,50]. Upon binding with ABA, *PYLs* interact with *PP2C* and inhibit *PP2C* by repressing phosphatase activity; in turn, the inhibition of *PP2C* results in the inactivation of ABA [51], which are core components of the ABA signaling pathway [51,52], allowing accumulation of phosphorylated downstream substrates and ABA transcriptional responses. Given that *PLY4* (upregulated) and *PP2C* (downregulated) were only detected as DEGs in sprouting-induced conditions (Figure 2C, Supplementary Figure S4) in this RNA-seq analysis, it is likely that *PYL4* interacted with *PP2C* in response to ABA, reducing the ABA level. As the balance of ABA/GA levels constitutes a vital regulatory mechanism to maintain and release seed dormancy in cereal crops [53], although there is no clear correlation between the ABA content and the tuber sprouting in different potato varieties [54], we speculate that it is possible that the change in ABA level was involved in sprouting growth, providing a reference on potential mechanisms initiating and inhibiting tuber sprouting growth for further study.

### 3.3. Regulation of the Cytokinin Homeostasis Response to Sprouting Growth

Cytochrome P450 monooxygenases *CYP735A* catalyzed the hydroxylation of iPRDP and iPRTP nucleotides to produce trans-CK [55,56] in the “Zeatin biosynthesis” pathway (Figure 2C), and the upregulated *CYP735A* indicates that CK biosynthesis signaling was activated in sprouting, agreeing with *CYP735A* being actively expressed in tuber sprouts [57]. Cis- to trans-isomerization was initially considered as a potential route to trans-CK, and cis-CK most likely plays a role in housekeeping to maintain a minimal CK activity [58,59]. *CISZOG* genes encode a stereo-specific O-glucosylation of cis-zeatin, and *UGT85A1* codes the CK O-glucosylation through O-glucosyltransferases, representing zeatin O-glucosyltransferease with a preference for trans-zeatin and substantially contributing to CK homeostasis [60,61]. *CISZOG* raises the possibility that cis-CK derivatives play a vital role in cytokinin homeostasis [62]. Cis-CK was also considered as a potato tuber dormancy regulator [63], and the upregulation of *CISZOG* and *UGT85A1* (Figure 2C) suggests that CK homeostasis plays a significant role in the developmental process of sprouting. Moreover, almost all cis-CKs are believed to be resistant to cytokinin dehydrogenase/oxidase (*CKX*), which is essential for mediating CK degradation [64]. However, no genes related to *CKX* were detected as DEGs in this study. These results suggest that CK homeostasis played a crucial role in GA-induced sprouting.

## 4. Materials and Methods

### 4.1. Plant Materials and Culture Conditions

The explants were propagated using in vitro single nodal segments (containing the axillary buds) with leaves excised and were incubated in the 400 mL culture bottles containing the MS medium [65], supplemented with 25 g/L sucrose and 4 g/L agar (referred to as the MS subculture medium) at 25 °C with cycles of 16 h of light and 8 h of darkness. The multiplication phase was routinely repeated every 4 weeks to obtain shoot explants for the research, using *Solanum tuberosum* L. subsp. *andigena*. For MT induction, a single nodal segment (ca. 1 cm long) containing an axillary bud was cut from shoot explants and then incubated on the MS subculture medium in the dark at 25 °C for two days to break the dormancy of the axillary bud. These single nodal segments were transplanted and further incubated in the dark at 20 °C for two weeks on the MS medium supplemented with 100 g/L sucrose, 4 g/L agar, and 20 μM ancymidol (Sigma-Aldrich, St. Louis, MO, USA) (called the MS tuberization medium). To examine the effects of plant hormones on MT sprouting, we transferred the 2-week-old MTs attached to the nodal stem (stemmed MTs, stMTs) to the MS subculture medium supplemented with 10 μM GA_3_ (Wako, Osaka, Japan), 10 μM 6-benzyladenine (a synthetic cytokinin, CK) (Wako, Osaka, Japan), both GA_3_ and CK (GACK), or with no additional plant hormones (untreated control), and then incubated them for 15 days in the dark at 20 °C. To explore the role of the nodal stem attached to the MT in sprouting, we detached the nodal stem from the stMTs, but the junction part remained attached to the MTs (destemmed MTs, deMTs). In addition, we cut the MTs in half along the equatorial plane and entirely removed the nodal stem (including the junction part with MTs) (MT halves, hMTs). Both deMTs and hMTs were cultured as described above. For each condition, 30 MTs were tested, and 3 biological replicates were collected simultaneously. The preparation methods for the MTs are summarized in Appendix A, Figure A1A.

### 4.2. Sample Preparation for RNA Extraction and Sequencing

MTs attached to the nodal stems (stMTs) and MT halves (hMTs) prepared under each hormone culture condition were incubated at 20 °C in the dark for 3 days. We selected samples on day 3 of culture to analyze the gene and profile responses to GA and CK in regulating tuber sprouting during the breakage of dormancy and the sprout outgrowth, due to the sprouting rate showing a non-linear increasing trend after day 3 of culture for stMTs and hMTs (Figure 1B,D), with a criterion of sprouting rate below 50%. Samples from 12 conditions were pooled for total RNA extraction in triplicate using the SDS-LiCl method [66] and treated with TURBO DNase (Invitrogen, Waltham, MA, USA), including apical bud tissues (about 100–120 mg) from stMTs or hMTs treated with GA, CK, GACK, or control (untreated), respectively, and nodal stem tissues (about 100–120 mg) from stMTs treated with GA, CK, GACK, or control. Sample (a total of 36) collection for RNA-seq analysis in detail on day 3 of culture is depicted in Appendix A, Figure A1B. Taking tissue samples was the first critical step in isolating quality RNA. All samples taken in this experiment were flash-frozen in liquid nitrogen and stored in a freezer at −80 °C until RNA extraction. Total RNA was judged to be of good quality based on absorbance measurements and electrophoresis and was then used for subsequent RNA sequencing (RNA-seq). RNA-seq using a NovaSeq 6000 platform (Illumina, San Diego, CA, USA) was conducted at Macrogen (Tokyo, Japan). Each sample returned between 40,134,914 and 85,943,080 total reads for 100 bp paired-end sequencing.

### 4.3. TPM Value Calculation and Differential Expression

Trimmomatic (version 2.13) [67] and the Cutadapt program [68] were used to trim adapter sequences and low-quality reads from the above. The obtained clean reads were mapped to the potato genome sequence of doubled monoploid *S. tuberosum* (PGSC_DM_v4.03) downloaded from the Spud DB Potato Genomics Resource, using the HISAT2 (version 2.0.1) [69]. The default settings were used for mapping. In total, 84.96-89.14% of reads per sample aligned to the transcriptome. The mapped reads were assembled and counted using StringTie (version 2.2.1) [70], and both the estimated raw count values and the normalized expression as transcript per million (TPM) for each gene were retrieved from the mapping. DESeq2 [71], running through the R (version 4.2.2), was then used to analyze differentially expressed genes (DEGs) between the two samples based on the raw counts (DEGs were calculated by comparing the gene expression between the exact type of tissues in the same conditions) with an absolute value of log2(FC) (|log2 fold change|) > 1 and a false discovery rate (FDR) < 0.05 selected as thresholds. Detected DEGs were then annotated against ENSEMBL release *S. tuberosum* SolTub_3.0 or a search tool for orthologous genes (g: Orth) in g: Profiler [72], Gene Ontology (GO), and KEGG. In this work, a *p* < 0.05 cutoff was taken after enrichment analysis to indicate significantly enriched GO functions and KEGG pathways [73] using DAVID Bioinformatics Resources. The gene expression profile was visualized using the ClusterProfiler 4.0 package through the R [74].

### 4.4. Module Construction Using WGCNA

The weighted gene correlation network analysis (WGCNA) was conducted using the WGCNA package in R [75]. The parameters were as follows: the soft threshold power was 14; the min module size was 30; and the merge cut height was 0.25 for a total of 6515 DEGs involved in treatments on day 3 of culture, with a TPM value > 1 in at least one of the samples to be identified from the 36 samples used for performing the co-expression network analysis in our work. The eigengene values were computed for each module to identify associated links with potato MT sprouting, with a visualization of the interaction network conducted via Cytoscape (version 3.10.0) [76].

### 4.5. Quantitative Analysis of Candidate Genes Involved in Potato Microtuber Sprouting

Based on transcriptome results, eight genes involved in the regulation of MT sprouting were selected for quantitative real-time polymerase chain reaction (qRT-qPCR) analysis to examine the transcriptomic profiles, with the primers listed in Table 2. Primer design was conducted based on the output results of Primer 3 (version 0.4.0): https://bioinfo.ut.ee/primer3-0.4.0/ (accessed on 28 August 2023). Oligonucleotides used for primers in RT-qPCR were synthesized by Fasmac Co., Ltd. (Atsugi, Japan). qPCR reactions were performed as follows: initial denaturation at 95 °C for 15 min, followed by 40 cycles, each with denaturation at 95 °C for 10 s and annealing at 60 °C for 30 s. Finally, melting curve analysis was conducted in a 65–95 °C temperature range. The samples from the three independent biological replicates were used for the analyses. The quantitative data of candidate genes were calculated using the 2^−∆∆Ct^ method [77].

### 4.6. Quantification and Statistical Analysis

In the case of the sprouting rate, data from three biological replicates were provided as means (±standard deviation); in the case of qPCR and TPM, data from three biological replicates were provided as means (±standard error). All data were subjected to analysis of variance (ANOVA) using GraphPad PRISM (version 10), and mean differences were tested for statistical significance using Tukey’s multiple comparisons with a 95% confidence level outcome.

## 5. Conclusions

In this study, to understand the role of CK and the nodal stem in GA-induced sprouting, we investigated the profiling of gene expression change at the transcriptome level using MTs (stMTs and hMTs) with treatment of GA, CK, GACK, or the untreated control. In our observation experiments, GA alone was sufficient to induce stMT sprouting but failed to induce hMT sprouting, However, CK co-application could compensate for the absence of the nodal stem, supporting GA-induced sprouting. RNA-seq analyses showed that DEGs associated with “Plant hormone biosynthesis and signal transduction” were detected as the hub genes in this study. In sprouting buds, genes related to the “CK-activated signaling pathway” and “CK biosynthesis process” were downregulated. However, genes related to “Zeatin biosynthesis”, such as *CYP735A* and *CISZOG,* were upregulated. Although GA treatment activated genes related to auxin, as is evident from genes related to the “auxin-activated signaling pathway” in response to sprouting, and even in sprouting-failed conditions, the lack of regulation of *IAA15*, *IAA22*, and *SAUR50* in GA-treated hMTs made the nodal stem or CK co-application very important for GA-induced sprouting. Furthermore, the ABA negative regulators, *PLY4* and *PP2C*, were only detected in sprouting conditions. These results made us conclude that the endogenous CK is involved in the nodal stem and plays a vital role in GA-induced sprouting by maintaining CK homeostasis to sustain the developmental processes in sprouting, which impacts the expression of these key genes.

There is still much to be learned about these genes relevant to the crosstalk among GA, CK, and auxin regarding the control of sprouting. More research on the mechanisms and the verification of genes involved in this sprouting process using overexpression, RNAi, or CRISPR/Cas9 systems needs to be conducted with both apical buds and nodal stems with a time course design, especially the timing of dormancy break. In addition, other factors, such as environmental factors, nitrogen supply, growth regulators, temperature, potato cultivar, and sucrose concentration, should be investigated with reasonable orthogonal experimental designs, given that global warming has become a significant issue for potato tuber secondary growth. This study provides further insight into the potential molecular mechanisms initiating and motivating tuber sprouting and sheds light on avoiding and reducing tuber sprouting, allowing us to address and optimize the methods for breeding and cultivating potatoes.

## Figures and Tables

**Table 1:** Summary of RNA-seq data generated from 36 samples.

Sample Name *	Clean Reads	Mapped Reads (%)	GC Content (%)	Q30 Bases Rate (%)
3-stMT-bd-Con_1	43,681,568	86.01	43.23	92.36
3-stMT-bd-Con_2	41,043,396	84.96	42.71	92.84
3-stMT-bd-Con_3	42,184,662	86.44	42.60	92.70
3-stMT-bd-GA_1	48,289,086	86.39	43.16	92.62
3-stMT-bd-GA_2	53,701,594	87.57	43.57	92.29
3-stMT-bd-GA_3	48,581,534	86.63	43.00	92.91
3-stMT-bd-CK_1	39,696,672	86.79	42.60	93.58
3-stMT-bd-CK_2	55,057,060	86.93	42.78	93.03
3-stMT-bd-CK_3	48,249,408	86.56	42.73	93.34
3-stMT-bd-GACK_1	54,286,264	86.61	43.15	92.98
3-stMT-bd-GACK_2	45,950,936	87.25	43.07	92.92
3-stMT-bd-GACK_3	45,743,028	87.76	43.44	93.39
3-stMT-st-Con_1	45,423,060	86.01	42.85	92.27
3-stMT-st-Con_2	53,654,956	84.96	42.91	93.00
3-stMT-st-Con_3	59,954,606	86.44	43.64	92.27
3-stMT-st-GA_1	53,629,136	86.39	43.25	92.78
3-stMT-st-GA_2	55,193,846	87.57	43.38	92.85
3-stMT-st-GA_3	49,795,808	86.63	43.49	92.97
3-stMT-st-CK_1	53,347,826	86.79	43.45	93.14
3-stMT-st-CK_2	54,308,396	86.93	43.13	93.25
3-stMT-st-CK_3	46,884,960	86.56	43.44	92.96
3-stMT-st-GACK_1	48,972,376	86.61	43.51	93.15
3-stMT-st-GACK_2	40,544,512	87.25	43.62	93.36
3-stMT-st-GACK_3	49,480,050	87.76	42.75	92.91
3-hMT-bd-Con_1	43,157,574	85.59	42.17	92.56
3-hMT-bd-Con_2	56,598,482	85.63	42.18	92.53
3-hMT-bd-Con_3	47,043,018	85.65	43.27	92.74
3-hMT-bd-GA_1	55,008,322	86.01	42.75	92.95
3-hMT-bd-GA_2	52,098,892	84.96	42.16	92.83
3-hMT-bd-GA_3	40,728,744	86.44	42.85	92.77
3-hMT-bd-CK_1	43,493,506	86.39	42.91	93.17
3-hMT-bd-CK_2	54,588,608	87.57	42.61	93.49
3-hMT-bd-CK_3	49,590,678	86.63	42.75	93.62
3-hMT-bd-GACK_1	44,376,982	86.79	42.82	93.23
3-hMT-bd-GACK_2	50,462,110	86.93	43.24	93.33
3-hMT-bd-GACK_3	46,772,868	86.56	43.83	93.27

* Sample name includes all tissues used to extract RNA; bd: apical bud tissue; st: nodal stem tissue; stMT: stemmed microtuber; hMT: microtuber half. Clean reads: number of reads remaining after filtering; Q30 bases rate: proportion of nucleotides with a quality value larger than 30 in the filtered reads.

**Table 2:** Primer sequences were used to amplify target genes. α-tubulin for housekeeping as an internal control.

Gene ID PGSC0003DMG-	Primer	Primer Sequence (5′ → 3′)	Length of Oligonucleotides/bp
α-tubulin	α-Tub-rt-Fd1	CAACAAGTGTTGCTGAGGTCT	21
	α-Tub-rt-Rv1	CAGCCTACATCATTGCTCAGT	21
402022640	*RR4*-Fd2	CCAACTCTTTCACCTTCACCA	21
	*RR4*-Rv1	TGGCTGATCATTTTGAGTCG	20
400003084	*RR9c*-Fd2	TGTTTGGAAGAAGGAGCTGAA	21
	*RR9c*-Rv1	CAAATGTTCTGCAAAAAGATGC	22
400028331	*CISZOG*-Fd2	TTCGGGATGGAACTCTTTTCT	21
	*CISZOG*-Rv2	TCCATCAATGTTCTCACACCA	21
400012669	*CYP735A*-Fd1	ATGAAACCACTGCCCTTTTG	20
	*CYP735A*-Rv2	CCTCAAATGCCATTCTTGGT	20
400001614	*SAUR50*-Fd1	TTGGCTATACCTTGCGATGA	20
	*SAUR50*-Rv1	CCCAGTAACGCTCGAGATTC	20
400001655	*SAUR71*-Fd2	TCACAATCTCCTGTTTTGAAGC	22
	*SAUR71*-Rv1	GCCCATATCATGATCGAACC	20
400020139	*IAA15*-Fd1	ACCTGGAACAGAGCCATCAT	20
	*IAA15*-Rv1	TTAAATAAGCCGCACCATCC	20
400011033	*PYL4*-Fd1	GACGGTGACGTCGGTACTTT	20
	*PYL4*-Rv2	ATTCCACAACGATCGTCTCC	20

## Data Availability

The data presented in this study are available on request from the corresponding author. The data are not publicly available due to [The submission of RNA-seq data to the SRA database is being processed. Since we are working on the subsequent study using this RNA-seq data, the data will be released on 31 December 2024].

## Supplementary Materials

The supporting information can be downloaded at: https://www.mdpi.com/article/10.3390/ijms242417534/s1.
