# Supplementary material for: Transcriptomic Analyses Reveal the Role of Cytokinin and the Nodal Stem in Microtuber Sprouting in Potato (Solanum tuberosum L.)
Source: Int J Mol Sci. 2023 Dec 15;24(24):17534. doi: 10.3390/ijms242417534 (PMC10743403; doi:10.3390/ijms242417534)
Supplement: Supplementary file 1 [file ijms-24-17534-s001.zip › ijms-2753479-supplementary/Zhang_IJMS_supplementary files/Xia. et.al,_IJMS_Supplemental_information_revised.pdf]

Supplemental information

Transcriptomic Analyses Reveal the Role of Cytokinin and the Nodal Stem in Microtuber Sprouting in Potato (*Solanum tuberosum* L.)

Xia Zhang, Fujino Kaien *, and Hanako Shimura

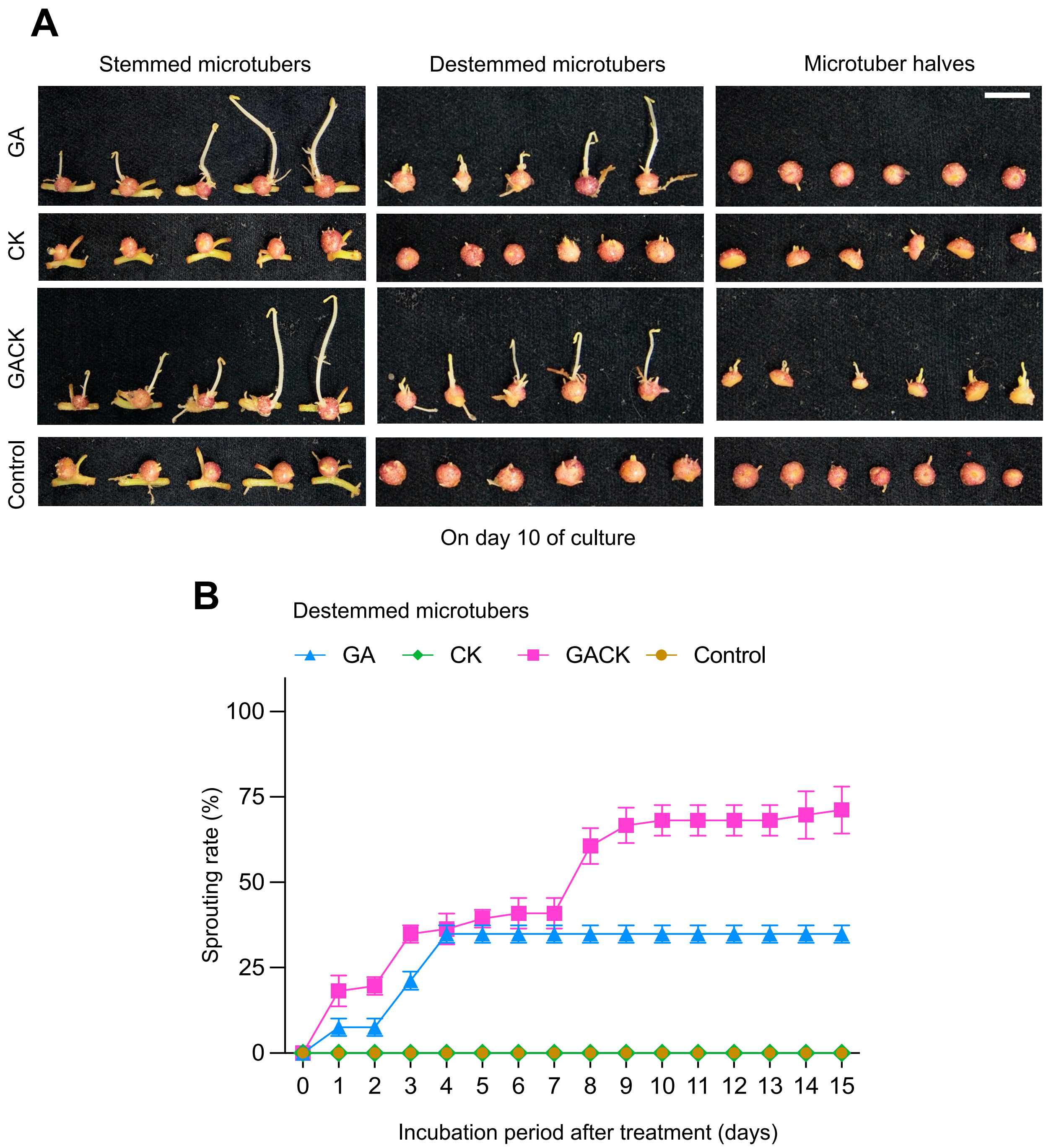

Figure S1. (**A**) Representative images of the visual appearance of sprouting for stemmed, destemmed microtubers, and microtuber halves on day 10 of culture, respectively. (**B**) Sprouting rate for destemmed microtubers, cultured with 10 µM GA_3 (GA), 10 µM benzyladenine (CK), or both (GACK), respectively. Incubation without phytohormone was set as the control (untreated). Each value represents the mean ± standard deviation of three replicates. Bar = 10 mm.

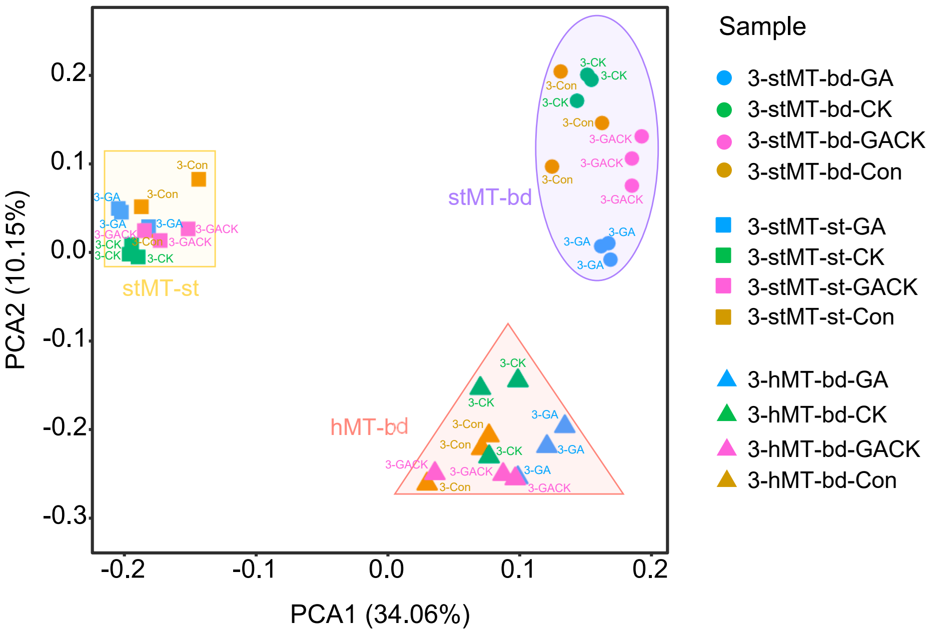

Figure S2. Principal component analysis (PCA) calculated TPM (Transcripts Per kilobase Million) values for RNA-seq data from 36 samples, visualized using the ggplot2 package in R. Genes included had a TPM over one for all replicates within our study (39,028 genes included in the analysis). Principal components 1 and 2 (PC1 and PC2) could represent 34.06% and 10.15% of all differences among apical bud tissues (bd) and nodal stem tissues (st) of stemmed microtubers (stMTs), and apical bud tissues (bd) of microtubers halves (hMTs), respectively. PCA plots represent the transcript levels from three hormone-treated groups (GA, CK, and GACK) and one untreated group (Con) on day 3 (3-) of culture. Each treatment has three biological replicates.

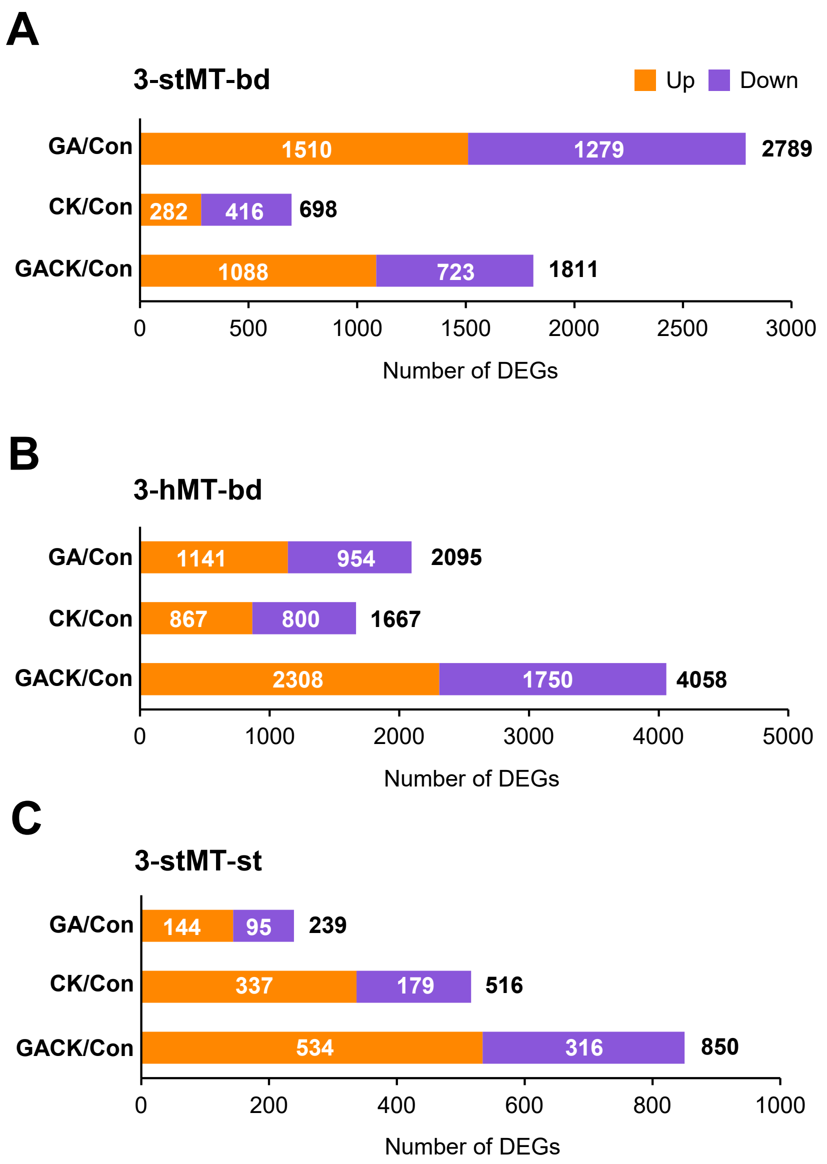

Figure S3. Numbers of the differentially upregulated (orange) and downregulated (purple) DEGs in the pairwise comparisons of phytohormone treatment (GA, CK, or GACK) with control (Con, untreated) in apical bud tissues (bd) of stemmed microtubers (stMTs) (**A**) and microtuber halves (hMTs) (**B**), and in the nodal stem tissue (st) of stMTs (**C**) on day 3 (3-) of culture. DEGs were calculated using the DEseq2 package in R. The total counts of DEGs were shown next to the bar.

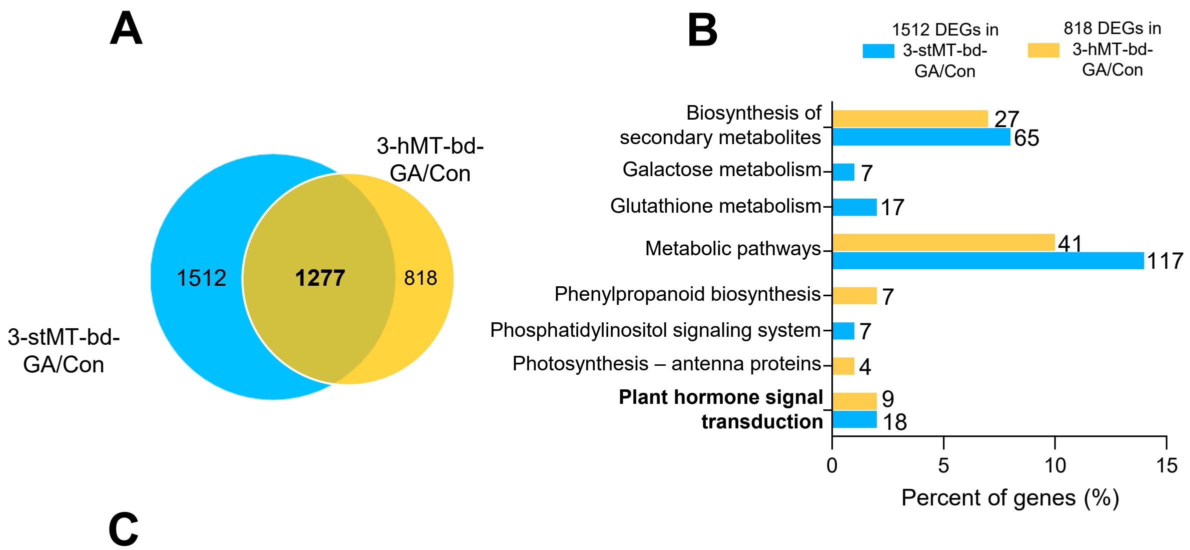

C

Plant hormone signal transduction

Gene ID PGSC0003DMG	Gene Description	Function	Unique 1512 3-stMT-bd GA/Con	Unique 818 3-hMT-bd GA/Con
400016317	IAA12	AUX/IAA gene family	1.57	not DEG
400020139	IAA15	AUX/IAA gene family	2.04	not DEG
400006108	IAA22	AUX/IAA gene family	1.19	not DEG
400021803	CH3.6	Auxin responsive CH3 gene family	2.09	not DEG
400024995	CH3.6	Auxin responsive CH3 gene family	-1.72	not DEG
400001601	SAUR	Auxin responsive SAUR family	3.42	not DEG
400001604	SAUR	Auxin responsive SAUR family	1.30	not DEG
400001614	SAUR	Auxin responsive SAUR family	2.36	not DEG
400001654	SAUR	Auxin responsive SAUR family	6.23	not DEG
400021991	GID1	Gibberellin receptor	-1.47	not DEG
400011033	PYL4	Abscisic acid receptor	1.55	not DEG
400015897	PYL4	Abscisic acid receptor	2.45	not DEG
400017514	PYL4	Abscisic acid receptor	1.14	not DEG
400002573	PP2C	Protein phosphatase 2C ABI2	-1.10	not DEG
400015853	EBF1/2	EIN3-binding F-box protein 1-like	-1.51	not DEG
400028694	EIN4	Ethylene receptor	-1.12	not DEG
400029834	NPR5	Regulatory protein	-1.08	not DEG
400005109	PR-1	Pathogenesis-related protein	2.45	not DEG
400003084	RR9c	Type-A response regulator	not DEG	1.24
400018579	PHP4	Phosphotransfer protein 4-like	not DEG	7.50
400003771	ARF5	Auxin response factor	not DEG	-1.15
400012261	ARF9	Auxin response factor	not DEG	1.40
400001668	SAUR	Auxin responsive SAUR family	not DEG	1.69
400029190	SAUR	Auxin responsive SAUR family	not DEG	1.66
400015667	JAZ	Jasmonate-zim-domain protein	not DEG	-1.19
400023678	TGA	bZIP transcription factor	not DEG	-1.02
400005116	PR-1	Pathogenesis-related protein	not DEG	-1.70

-1.72 7.50

Figure S4. DEG Comparison of apical bud tissues (bd) between stemmed microtubers (stMTs) and microtuber halves (hMTs) on day 3 (3-) of culture treated with GA. (**A**) A Venn diagram to visualize the similarities and differences of DEGs between 3-stMT-bd-GA/Con and 3-hMT-bd-GA/Con, and they harbored 1515 and 818 unique DGEs, respectively. (**B**) The comparison of enriched KEGG pathways for each harbored unique DEGs using DAVID ($p < 0.05$), visualized using GraphPad PRISM. Counts of DEGs included in each KEGG pathway are shown next to the bar in the graph. (**C**) The table shows genes in the "Plant hormone signal transduction" pathway for each harbored unique DEGs group, with a heatmap showing gene expression levels as log2FC. Orange represents upregulation, and purple represents downregulation compared to control (untreated).

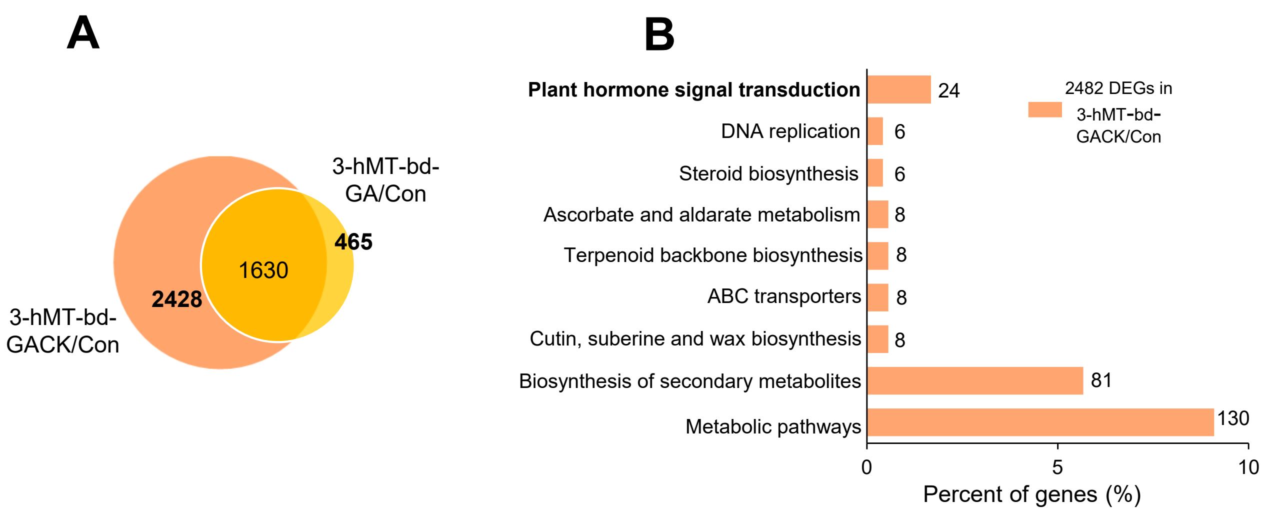

C

Plant hormone signal transduction

Gene ID PGSC0003DMG	Gene Description	Function	Unique 2482 3-hMT-bd GACK/Con	Unique 465 3-hMT-bd GA/Con
400001589	AUX1	Auxin influx carrier	1.76	not enriched pathways
400019182	AUX1	Auxin influx carrier	2.51	
400023205	AUX1	Auxin influx carrier	2.65	
400020139	IAA15	Aux/IAA gene family	4.13	
400006108	IAA22	Aux/IAA gene family	1.39	
400024997	GH3.6	Auxin responsive GH3 gene family	1.60	
400026186	GH3.6	Auxin responsive GH3 gene family	-1.38	
400001601	SAUR	Auxin-responsive SAUR family	2.91	
400001613	SAUR	Auxin-responsive SAUR family	5.45	
400001614	SAUR	Auxin-responsive SAUR family	3.28	
400001653	SAUR	Auxin-responsive SAUR family	-6.17	
400001654	SAUR	Auxin-responsive SAUR family	4.95	
400001655	SAUR	Auxin-responsive SAUR family	2.93	
400022233	SAUR	Auxin-responsive SAUR family	-2.05	
400028254	SAUR	Auxin-responsive SAUR family	-2.64	
400029463	HK4	Cytokinin receptor histidine kinase	1.36	
402022640	RR4	Type-A response regulator	2.17	
400029839	RR9b	Type-A response regulator	1.96	
400029852	RR9d	Type-A response regulator	3.96	
400027597	RR17b	Type-A response regulator	9.67	
400021991	GID1	Gibberellin receptor	-1.27	
400011033	PYL4	Abscisic acid receptor	1.40	
400015442	BKI1	Negative regulator of brassinosteroid signaling	-1.54	
400013975	TCH4	Xyloglucan endotransglycosylase	-1.62	

-6.17 9.67

Figure S5. DEG Comparison of apical bud tissues (bd) between GACK- and GA-treated microtuber halves (hMTs) on day 3 of culture. (**A**) A Venn diagram to visualize the similarities and differences of DEGs between 3-hMT-bd-GACK/Con and 3-hMT-bd-GA/Con, and they harbored 2428 and 465 unique DGEs, respectively. (**B**) Comparison of enriched KEGG pathways for each harbored unique DEGs using DAVID ($p < 0.05$). Counts of DEGs included in each KEGG pathway are shown next to the bar in the graph. (**C**) The table shows genes in the "Plant hormone signal transduction" pathway for each harbored unique DEGs group, with a heatmap showing gene expression levels as $\log_2$ FC. Orange represents upregulation, and purple represents downregulation compared to control (untreated).

A

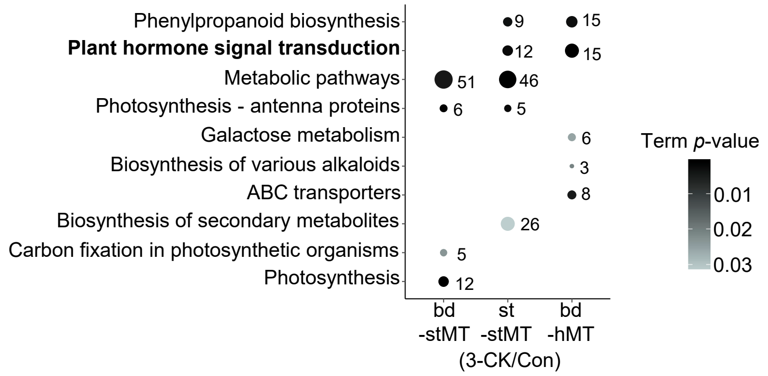

B

Plant hormone signal transduction

Gene ID PGSC0003DMG	Gene Description	Function	Sprouting-failed 3-stMT-bd CK/Con	Sprouting-failed 3-stMT-st CK/Con	Sprouting-failed 3-hMT-bd CK/Con
400003084	RR9c	Type-A response regulator	not enriched pathways	1.29	2.21
400002945	RR9a	Type-A response regulator		not DEG	1.89
400029839	RR9b	Type-A response regulator		not DEG	1.22
400029852	RR9d	Type-A response regulator		not DEG	2.18
402022640	RR4	Type-A response regulator		not DEG	1.21
400027597	RR17b	Type-A response regulator		not DEG	8.17
400029463	HK4	Cytokinin receptor histidine kinase		not DEG	1.17
400018579	PHP4b	Phosphotransfer protein 4-like		-1.87	6.87
400001589	AUX1	Auxin influx carrier		1.03	not DEG
400023205	AUX1	Auxin influx carrier		not DEG	1.83
400001589	AUX1	Auxin influx carrier		not DEG	1.48
400006093	IAA23	AUX/IAA gene family		-1.28	not DEG
400029339	IAA26	Aux/IAA gene family		not DEG	2.86
400019274	GH3.6	Auxin responsive CH3 gene family		1.06	not DEG
400024978	GH3.6	Auxin responsive CH3 gene family		1.31	not DEG
400026186	GH3.6	Auxin responsive GH3 gene family		not DEG	-1.63
400021803	GH3.6	Auxin responsive GH3 gene family		not DEG	-1.86
400030234	SAUR	Auxin-responsive SAUR family		-1.07	-1.26
400004786	SAUR	Auxin-responsive SAUR family		-1.23	not DEG
400022233	SAUR	Auxin-responsive SAUR family		-1.86	-2.08
400001655	SAUR	Auxin-responsive SAUR family		-1.25	not DEG
400035783	SAUR	Auxin-responsive SAUR family		not DEG	1.36
400028254	SAUR	Auxin-responsive SAUR family		not DEG	-3.17
400003673	SAUR	Auxin-responsive SAUR family		not DEG	2.01
400001653	SAUR	Auxin-responsive SAUR family		not DEG	-3.78
400026010	SAUR	Auxin-responsive SAUR family		not DEG	-1.08
400001643	SAUR	Auxin-responsive SAUR family		not DEG	-5.49
400017186	ETR	Ethylene receptor 2-like		1.09	not DEG
400017231	ERF1	Ethylene-responsive transcription factor 1B-		1.14	not DEG
400015667	JAZ	Jasmonate-zim-domain protein		not DEG	-2.51
401018740	TCH4	Xyloglucan endotransglycosylase		not DEG	-1.01
400013975	TCH4	Xyloglucan endotransglycosylase		not DEG	-1.24
400007110	TGA	bZIP transcription factor		not DEG	-1.72
400005116	PR-1	Pathogenesis-related protein		not DEG	-2.02

-5.49 8.17

Figure S6. Comparison of enriched KEGG pathways for DEGs among apical bud tissues (bd) and the nodal stem tissues (st) of stemmed microtubers (stMTs) and bd of microtuber halves (hMTs), respectively, on day 3 of culture with CK treatment compared with control (untreated). (**A**) The comparison of enriched KEGG pathways among three comparisons using DAVID ($p < 0.05$), visualized using the ggplot2 package in R. Counts of DEGs included in each KEGG pathway are shown next to the plot. (**B**) The table shows genes included in "Plant hormone signal transduction" for each comparison, with a heatmap showing gene expression levels as log2 FC. Orange represents upregulation, and purple represents downregulation compared to control (untreated).

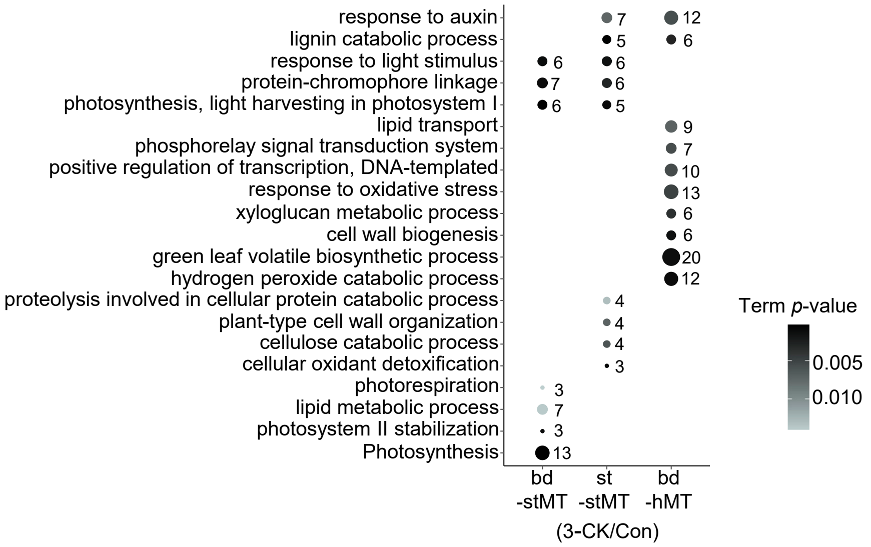

Figure S7. Comparison of enriched GO terms (top ten) in biological processes for DEGs among apical bud tissues (bd) and the nodal stem tissues (st) of stemmed microtubers (stMTs) and bd of microtuber halves (hMTs), respectively, on day 3 of culture with CK treatment compared with control (untreated) using DAVID ($p < 0.05$), visualized using the ggplot2 package in R. Counts of DEGs included in each GO term are shown next to the plot.
